# Physicians' attitude towards tobacco dependence in a private hospital in the city of São Paulo, Brazil

**DOI:** 10.1590/S1679-45082013000200004

**Published:** 2013

**Authors:** Alessandra Maria Julião, Ana Luiza Lourenço Simões Camargo, Vanessa de Albuquerque Cítero, Mara Fernandes Maranhão, Alfredo Maluf, Ângela Tavares Paes, Milton Glezer, Miguel Cendoroglo, Cláudio Schvartsman

**Affiliations:** 1 Hospital Israelita Albert Einstein, São Paulo, SP, Brazil

**Keywords:** Smoking/therapy, Health acknowledgment, attitudes, practice, Tabacco

## Abstract

**Objective:**

To investigate how often physicians identify and treat tobacco dependence and whether characteristics as gender, age, marital status, medical specialty and smoking status can influence their attitude towards this question.

**Methods:**

A cross-sectional study was performed on 515 physicians working in a private hospital in São Paulo, Brazil, using a confidential voluntary questionnaire sent and answered electronically.

**Results:**

We found that 89% of physicians who answered the research questionnaire often or always asked their patients about smoking habits, but only 39% often or always treated patients' tobacco dependence. In our sample, 5.8% of individuals were current smokers. Tobacco dependent physicians provided less treatment for smoking dependence compared with those who had never smoked, or were former smokers. Being a clinician was associated with higher probability to treat tobacco dependence.

**Conclusion:**

Physicians should not only address patients' smoking habits but also provide treatment whenever tobacco dependence is diagnosed. To understand physicians' attitude towards smoking may help to develop strategies to stimulate patients' treatment. The development of smoking cessation programs meant specifically for physicians may also be a strategy to enhance patients' treatment.

## RESUMO

**Objetivo:**

Observar se os médicos identificam e se tratam o tabagismo de seus pacientes, bem como investigar possíveis associações entre essas práticas e os seguintes fatores: gênero, idade, estado civil, especialidade médica e tabagismo do médico.

**Métodos:**

Estudo transversal realizado com 515 médicos atuantes em um hospital particular da cidade de São Paulo, por meio de um questionário confidencial enviado e respondido por *e-mail*.

**Resultados:**

Dentre os médicos, 89% frequentemente ou sempre pergunta se seus pacientes fumam, enquanto apenas 39% respondeu que frequentemente ou sempre trata essa condição. A taxa observada de tabagismo entre os médicos foi de 5,8%. Entre os fatores estudados, observou-se que o tabagismo do médico está associado a menor frequência de tratamento do tabagismo dos pacientes e que ser de especialidade clínica é um fator associado a maior probabilidade de tratar o tabagismo dos pacientes.

**Conclusão:**

Além de identificar o tabagismo dos pacientes, é fundamental que os médicos ofereçam tratamento para essa condição. O conhecimento da atitude dos médicos a respeito da dependência de tabaco pode auxiliar no desenvolvimento de estratégias que resultem em aumento da oferta de tratamento aos pacientes. O desenvolvimento de programas de tratamento para médicos tabagistas também pode ser uma medida com impacto positivo na oferta de tratamento aos pacientes.

## INTRODUCTION

According to the World Health Organization^(1)^, smoking is the most preventable cause of death throughout the world. In Brazil, the average prevalence of tobacco use in the general population is 16.1%^(2)^. When compared to prevalence rates found in previous studies^(3-5)^, this rate indicates a significant smoking reduction, which is a result of efforts such as campaigns to raise awareness of tobacco hazards and tobacco-free spaces in order to control this epidemic.

The attitude of healthcare professionals, particularly physicians, is an important factor to control tobacco dependence^(6)^. A multicenter survey with general practitioners and family physicians from 16 countries showed that physicians who smoked had a lower likelihood of addressing tobacco use during consultation of patients^(7)^. Other studies suggested that the condition of being a smoker is one of the factors that influence how physicians approach smoking patients^(8,9)^. According to Kawakami et al., professionals who smoke are less willing to collect data on history of tobacco use of their patients, besides being less motivated to provide counseling^(8).^

Little is known about physicians attitude on addressing patients smoking habits in Brazil^(6)^. Hence, we conducted a survey with physicians from a private hospital in the city of São Paulo (SP), Brazil. This study was motivated by the need of the hospital implementing several institutional measures, such as a tobacco-free environment throughout the hospital and training of the nursing team to identify smoker inpatients and perform a counseling intervention.

## OBJECTIVE

To investigate how often physicians identify and treat tobacco dependence and how characteristics such as gender, age, marital status, medical specialty and smoking status can influence their attitude towards this matter.

## METHODS

A cross-sectional study was conducted in October 2009 on physicians working at *Hospital Israelita Albert Einstein*, a private and non-profit general hospital in São Paulo, Brazil.

All 5,826 credentialed physicians were invited to participate in the survey. An explanatory electronic message (e-mail), with a self- completion form attached was sent to physicians. Of this total, 515 individuals sent the complete questionnaire anonymously. Participation in the survey was considered as an agreement to be enrolled in the study, as explained in the text of the e-mail.

The survey instrument was composed of questions addressing:

– sociodemographic data (genre, age and marital status);
– specialty;
– how often the physician asks patients about and treats their tobacco dependence;
– data about the physician smoking status (whether or not smoker).

For smoker physicians, the Fargeström Questionnaire was applied, and the motivational stage to quit smoking was evaluated.

The Fagerström Test for nicotine dependence^(10)^ is a commonly used for measuring of tobacco dependence for smokers. The cut-offs used for this instrument were: low dependence – 0 to 4; medium dependence – 5 to 7; high dependence – 8 to 0. This questionnaire was validated in Brazil by Carmo and Pueyo^(11)^.

The motivational stage for smoking cessation was assessed by one question about smoking with five categories of answers, each one corresponding to a motivational stage, as following:

1. precontemplative: individuals are either unaware of the damaging effects of tobacco or recognize smoking as a problem;
2. contemplative: individuals are aware of smoking's hazardous effects, but there is no interest in changing tobacco habits;
3. preparation stage: individuals want to change their behavior and are making plans to do so;
4. action: the change in behavior is accomplished;
5. maintenance: individuals focus on maintaining the abstinence.

The study was submitted and approved by the hospital's Ethics Committee (CAAE: 0054.0.028.000-09).

Data were assessed by descriptive analysis, with presentation of the categorical variables in simple and relative frequencies (percentages) of the numerical variables in means, medians, standard deviations, minimum and maximum values. In the comparative analysis, the differences of categorical variables were verified using the χ^2^ square test. As for comparisons of numerical variables, the Student's t test or the non-parametric Mann-Whitney test was used for independent samples. The significance level adopted was 0.05.

## RESULTS

Of the 515 physicians who participated in the survey, 344 (66.8%) were males. The mean age was 45.3±11.2 years (26 to 81 years). Regarding marital status, 79% (n=407) were married or lived with a partner, 12.8% (n=66) were single and 8.2% (n=42) were separated, widow/er or divorced.

The medical specialties were grouped into four categories, as shown in table 1. Anesthesiology was considered separately since the question about smoking is usually included in the pre-anesthetic evaluation. The group 4 gathered the specialties with theoretically more restricted contact with patients because they do not involve appointments, like radiology, pathology, preventive and social medicine and also doctors with activities restricted to hospital administration.

**Table 1:** Grouping of medical specialties

Specialty	n (%)
Group1: Anesthesiology	58 (11.3)
Group 2: Clinical specialties	169 (32.8)
Group 3: Surgical specialties	250 (48.5)
Group 4: Other specialties	38 (7.4)

Those who reported the current use of tobacco or having quitted smoking for less than 2 months were considered smokers and those who declared stopping smoking for over 2 months were deemed as former smokers. Results are displayed in table 2.

**Table 2:** Physicians' smoking habit

Physicians' smoking habit	n (%)
Never smoked	365 (70.9)
Former smoker	120 (23.3)
Smoker	30 (5.8)

Among the physicians who smoke, the evaluation of motivational stage for smoking cessation demonstrated that 16.7% (n=5) were in pre-contemplation stage, 20.0% (n=6) in contemplation, 36.7% (n=11) preparation for action and 26.7% (n=8) in action.

Smokers were classified concerning severity of nicotine dependence according to the Fargeström Scale. We found that 20% (n=6) presented moderate dependence (score between 5 and 7) and 80% (n=24) presented mild dependence (score lower than 4). No smoker of the sample was scored above 7, which characterized severe dependence. The severity of nicotine dependence and motivational stage were not included in the analysis because of the low rate of smokers among participants of the study.

There were no associations between physicians' sociodemographic characteristics and physicians' smoking status.

It was observed that most participants often or always asked their patients about smoking habits (89.3% of physicians, n=460). However, 61% (n=314) answered they never or seldom prescribed treatment, and only 39% (n=201) reported to treat smoking always or frequently.

Among the specialties grouped as previously described, 100% of the anesthesiologists answered that they always ask patients about smoking. For this reason, this category was not included in the analysis of associations with approach and treatment.

There were no statistically significant associations between the frequency in which physicians ask patients about smoking and the participants' sociodemographic data or physicians' smoking status.

Excluding Group 1 (anesthesiologists) and Group 4 (other specialties) from the sample, and comparing clinicians and surgeons as to addressing smoking, we observed that surgeons approached the issue more often than clinicians, though this difference was not statistically significant, but indicated a trend for greater approach by surgeons (p=0.057) – among clinicians, 85.2% (n=144) always or often asked patients if they smoke, while the frequency among surgeons was 91.2% (n=228).

With respect to the attitude towards treatment, there was no association with physician's sociodemographic characteristics. The physician's specialty and smoking status were the factors associated with the likelihood of physicians treating patient's tobacco dependence. We found that clinicians treated smoking more frequently than surgeons. Among clinicians, 55.0% (n=93) always or frequently treated, as compared to 35.2% (n=88) of surgeons. This difference was statistically significant. The results of association to physician's smoking status are shown in table 3. These two associations remained statistically significant in a logistic regression model (Table 4).

**Table 3:** Physicians’ smoking and likelihood of a physician treating the smoking of his/her patient

Physicians report treating patient's smoking	Smokers *versus* never smoked		Smokers *versus* former smokers	
Never or seldom treats – n (%)	25 (83.3)	220 (60.3)	25 (83.3)	69 (57.5)
Always or often treats – n (%)	5 (16.7)	145 (39.7)	5 (16.7)	51 (42.5)
p value	0.012		0.009	

**Table 4:** Logistic Regression Model considering physicians' attitude towards treatment as the dependent variable

Variables	Coefficient (B)	df	Sig	Exp (B)	95% CI for Exp (B)	
					Lower	Upper
Specialty*	0.859	1	<0.001	2.36	1.56	3.57
Smoking status (smokers *versus* former smokers)	1.067	1	0.04	2.90	1.01	8.33
Smoking status (smokers *versus* never smoked)	1.08	1	0.05	2.94	0.989	8.761
Constant	-1.939	1	0.01	0.144		

* only clinicians and surgeons were considered

df: degrees of freedom; 95%IC: 95% confidence interval.

## DISCUSSION

We observed a low prevalence of current smokers and a high prevalence of ex-smokers in our sample. This study demonstrated that no sociodemographic characteristics of physicians had an impact on frequency of addressing and treating smoking of their patients. No other variable showed association with frequency that physicians ask patients if they smoke. On the other hand, to be a clinician and a non-smoker (including non-smoker and former smoker physicians) were features associated to highest frequency of treatment of smoker patients.

An important finding was the high frequency that physicians ask their patients about smoking, since the majority of the physicians reported asking patients routinely. It is well- known that identifying smoking habits of patients and systematically inquiring about it when collecting the patient's history are crucial. In a study carried out in the United States by Ferketich et al.^(12)^, over 85 thousand medical records were analyzed from 2001 to 2004, and in 32% of them there was no information about tobacco dependence of patients. Among variables that may influence the attitude of physicians in this aspect, the authors mention availability of time, acknowledgement smoking as a health problem, knowledge of healthcare professionals about treatment methods, and the importance of providing counseling for smoking cessation.

The physician's specialty and some characteristics of physicians, such as whether they are smoker or not, have also been mentioned as possible factors that could influence the attitude of the professionals towards smoking, although there are few studies on the topic and they present controversial results^(7-9,13)^. According to a recent cross-sectional study^(14)^ that evaluated professionals of nine different clinical specialties, physicians document more often and with more details the clinical history data with greater relevance for diagnoses related to their field of expertise. In this study, asking about tobacco use was more frequent among cardiologists and gastroenterologists, while greater details about patient's smoking patterns were found among pneumologists. In our research, specialties were grouped into categories, and were not assessed separately.

The fact that surgeons inquired more often about tobacco use may be due to the impact of smoking in complications after surgical procedures^(15)^. Unfortunately, no other studies were conducted in Brazil to be compared with our results. Nonetheless, it is important to consider that although surgeons tend to inquire more about tobacco use, they often provide significantly less treatment to this condition than clinicians. This finding allows us to assume that many patients identified as smokers do not receive any kind of intervention for smoking cessation. Hence, the issue of the impact of the medical specialty on approach and treatment of tobacco dependence deserves further studies. Research in this field could provide new data to support strategic planning of educational measures for physicians, according to the priorities of each specialty. Our study results' may suggest that surgeons should be encouraged not only to ask about smoking habits but also to provide smoking treatment for their patients. At the same time, clinicians should be stimulated to systematically address tobacco dependence in clinical practice, once they seem to be more prompted to treat the patient whenever they identify the smoking condition.

Concerning physicians' smoking status, our study demonstrated that being a smoker has no impact on addressing tobacco dependence, but it decreases the probability of delivering treatment. Physicians who smoke provide less treatment than physicians who have never smoked or are former smokers. This data corroborates with some in literature about this topic^(7-9)^, but not with all^(16)^. Some studies indicate less availability and motivation among physicians who smoke for counseling on smoking cessation^(14)^. In a study performed by Ulbricht et al.^(9)^, 609 patients were selected for a control group (called “care-the-usual control condition”) and 402 patients for the intervention group (brief counseling). The physicians who took part in this study were trained for 2 hours a week before the intervention. Patients were assessed five times: baseline, 6, 12, 18 and 24 months. The status of being a non-smoker physician had a positive effect in withdrawal measures in the long run (4 weeks and 6 months) in the group receiving counseling. Results suggested that benefits of an intervention, such as brief counseling, were even greater when provided by a non-smoker physician.

Our study had the merit of investigating this subjected for the first time in Brazil, and until the end of this investigation no Brazilian studies addressing this topic were found. We are aware of the limitations in this research that should be taken into account. In this sense, we acknowledge that our sample may not be representative of the general population of physicians working at the hospital where this study was conducted. Moreover, frequency of referral of smoker patients to treatment by other professionals was not investigated and comparisons among several medical specialties could not be performed. For this reason, we decided to gather specialties in broader areas.

## CONCLUSION

Systematically inquiring about smoking habit is crucial. However, physicians should not only address tobacco dependence but also provide treatment whenever this condition is diagnosed.

Understanding physicians' attitude towards tobacco dependence may help to develop strategies to stimulate treatment for smoking cessation that take into account specialties different needs in order to perform a better approach of the patient smoking condition. The development of smoking cessation programs meant specifically for physicians may also be a strategy to enhance patients' treatment, particularly because non-smoker physicians seem to treat smoking more frequently.
